# Analysis of Pelletizing of Granulometric Separation Powder from Cork Industries

**DOI:** 10.3390/ma7096686

**Published:** 2014-09-18

**Authors:** Irene Montero, Teresa Miranda, Francisco José Sepúlveda, José Ignacio Arranz, Sergio Nogales

**Affiliations:** Department of Mechanical Engineering, Energy and Materials, Industrial Engineering School, University of Extremadura, Av. Elvas s/n, 06006 Badajoz, Spain; E-Mails: tmiranda@unex.es (T.M.); fsepulveda@unex.es (F.J.S.); jiarranz@unex.es (J.I.A.); senogales@alumnos.unex.es (S.N.)

**Keywords:** cork, waste, pellets

## Abstract

Cork industries generate a considerable amount of solid waste during their processing. Its management implies a problem for companies that should reconsider its reuse for other purposes. In this work, an analysis of pelletizing of granulometric separation powder, which is one of the major wastes in cork industries and which presents suitable properties (as an raw material) for its thermal use, is studied. However, its characteristic heterogeneity, along with its low bulk density (which makes its storage and transportation difficult) are restrictive factors for its energy use. Therefore, its densified form is a real alternative in order to make the product uniform and guarantee its proper use in boiler systems. Thus, the cork pellets (from granulometric separation powder) in the study met, except for ash content specification, the specifications in standard European Norm EN-Plus (B) for its application as fuel for domestic use.

## 1. Introduction

Cork is the name given to cork tree’s bark (*Quercus suber*), which is a tree that is mainly found in the occidental Mediterranean. Cork is used in multiple applications, both in industry and building, emphasizing wine bottling. Indeed, 70% of world-wide production is devoted to cork stoppers.

It is estimated that there is around 2.5 million hectares of cork tree forests in the world, mainly located in Portugal (32%), Spain (27%), Algeria (17%) and Morocco (14%). Spain is also the second world-wide cork producer (with 23% total production) just after Portugal [1].

The manufacturing of cork is oriented to natural cork stoppers, being the central point of the rest of the related products and elements. The process starts with cork planks boiling in hot water for an hour, with the aim of increasing the thickness, flexibility and elasticity. Afterwards, plank classification is carried out, assigning a destination according to the quality and thickness. Planks that are valuable for cork stopper (or natural cork disks) manufacturing are sent to finishing industries, where these elements are obtained by direct perforation. The remaining (without enough quality for cork stopper manufacturing) are set aside for granulated cork industries, where they are previously ground for final agglomeration [2].

As a consequence, cork industries generate a considerable amount of solid wastes, making their proper management necessary. Major wastes, grinding powder and granulometric separation powder, are produced in granulated cork industries, representing 51% and 46% of the total solid waste [3]. Grinding powder is obtained after cork plank grinding, representing a high ash percentage that makes it difficult to be used in combustion process. Granulometric separation powder obtained from density separation presents better characteristics as a fuel, being the selected waste in this work for its densification study.

Cork waste energy use has been studied by several authors. Gil [4] carried out a physical and energy characterization of different kinds of cork powder, whose term used to include a group of wastes from transformation activities. Rojas *et al.* [5] suggested a selective storage of wastes for briquette manufacture and their following use as fuels in cork industries. 

In recent years, manufacturing and commercialization of densified biomass products have increased considerably, being a real alternative to fossil fuels and, therefore, making raw material (mainly wastes) valuable. Research concerning biomass densification has been diversified in two trends: one focuses on mechanism studies in order to optimize the densification process and its parameters (moisture, pressure, temperature, *etc.*) [6,7,8,9], and another based on the use of other biomass wastes, individually or jointly, as in the case of wastes from the cork industry.

Thus, Montero *et al.* [10] studied, separately, the different wastes that were generated, pointing out their main attributes (both in the original product and its respective densified), so as to make it possible to be used for a specific use.

Nunes *et al.* [11] densified cork wastes with different granule sizes, obtaining similar results to the pelletization of forest residues. Arranz [12] produced different pellets derived from Pyrenean oak, olive and grape pomace and cork powder mixtures, highlighting the higher high heating value and bulk density in the samples as pomace content was increased in Pyrenean oak mixtures. Mediavilla *et al.* [13] produced pellets by mixing cork wastes and grape pomace, optimizing the pelletizing process and reducing the total energy that is required for the process.

Nonetheless, no specific works about granulometric separation powder were found in the literature. Thus, most studies refer to cork residues in general, without distinguishing between them nor specifying their origin, with the subsequent inconvenience related to the fact that considerable differences in their properties might be found, depending on the stage or process in which they are generated (even within the same waste typology). 

This way, the aim of this work was to study the possibilities of different kinds of granulometric separation powder, with different grain sizes and physical characteristics. Next, pelletizing assays (with different mixtures, in order to assess both the need of pre-treatments and the effect of the resulting mixtures on the process) were carried out. Provided that granulometric separation powder is related to a specific agglomerated product manufacturing, pelletizing from waste mixtures, it would become independent from the demands of specific products, avoiding previous classifications or separate storage.

In this way, obtaining a high-quality densified product in these terms could make its use possible in domestic facilities or in service industries, its added value being increased considerably. 

With these premises, different pelletizing assays have been carried out in this research, combining different kinds of granulometric separation powder, with the aim of assessing its possible thermal use in real systems.

## 2. Materials and Methods

### 2.1. Wastes

Granulometric separation powder is generated in granulated cork industries. After an initial grinding of cork planks and their following sieving, a selection regarding their density was done. This task is carried out by slightly-inclined and vibratory boards, causing a regular movement in cork grains. This effect, along with air application, separates granulated cork (less dense) from the rest of the dense compounds (top and bottom of the planks), which constitute granulometric separation powder waste, or rejection powder, and lack the required qualities for being used in agglomeration industries.

**Figure 1:**
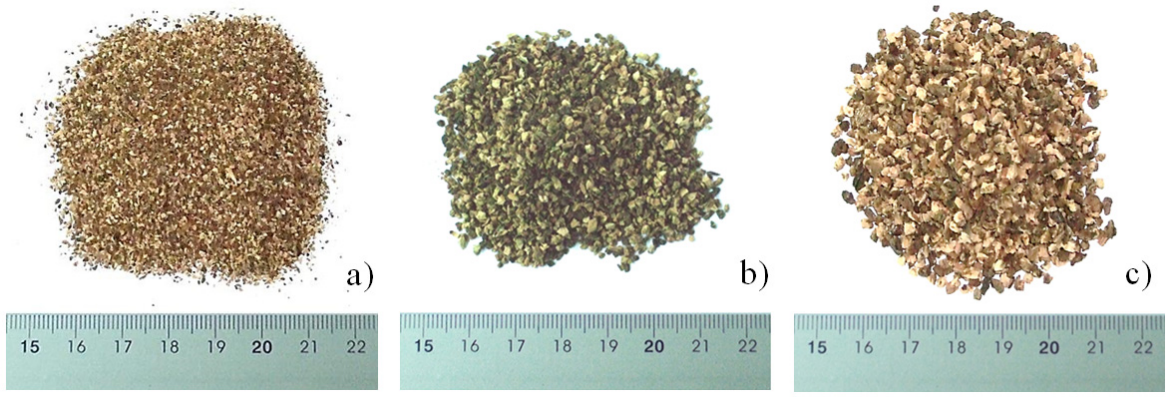
(**a**) 0.5–1 mm-sized powder; (**b**) 1–2 mm-sized powder; (**c**) 2–3 mm-sized powder.

There are different rejection sizes. For cork, the most common grain size ranges are 0.5–1 mm, 1–2 mm and 2–3 mm, the nomenclature of which refers to the grain size limit values. Those granulometries were used in the present work (Figure 1).

The selected samples were collected from a granulated cork industry in the southeast of Spain. For each waste, 150–200 kg was selected and transported in bags to our facilities in the Department of Mechanical Engineering, Energy and Materials (Industrial Engineering School, University of Extremadura, Badajoz, Spain) for their study. A small amount of sample (5 kg) was placed in hermetic bags for characterization assays. The remaining was used for pelletizing test. 

### 2.2. Waste Characterization

Moisture assays were carried out by a balance (accuracy ± 0.0001 g) and a stove (at 105 °C). The granulometric distribution was done by a siever (Restsch AS 200 basic, Retsch–Solutions in Millin & Sieving, Haan, Germany). For the ultimate analysis, an elemental analyzer (Eurovector EA 3000, Eurovector SpA, Milan, Italy) was used. For volatile matter and ash content percentage, an oven was used, achieving 900 and 550 °C, respectively. Fixed carbon content was calculated by subtracting the latter percentages from 100. A high heating value (*HHV*) was obtained by using a calorimeter (IKA C2000 Basic, Ika-Werke GmbH&Co, Staufen, Germany).

Finally, the low heating value (*LHV*) and energy density (*D_E_*) were determined through Equations (1) and (2), with *H* being hydrogen content (dry basis, db) and bulk density (*D_B_*, kg/m^3^ wet basis, wb).

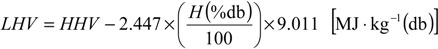
(1)

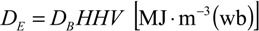
(2)


Moisture was determined by the standard UNE-EN 14772-2 [14]. Bulk density was determined by the standard UNE-EN 15103 [15]. Granulometry was defined by the standard UNE-EN 15149-2 [16]. Ultimate analysis was performed by the standard UNE-EN 15014 [17], for carbon, hydrogen and nitrogen, and the standard UNE-EN 15289 [18], for sulfur. Volatile matter and ash content were determined by the standard UNE-EN 15148 [19] and UNE-EN 14775 [20], respectly. And finally, heating value was calculated by the standard UNE-EN 14918 [21].

Concerning the three raw materials, characterization was complete, whereas for mixtures, only moisture and bulk density was obtained.

### 2.3. Sample Selection

Conditioning tasks (grinding, sifting, drying, *etc.*) of the wastes in the study were not considered, due to the fact that they presented, initially, the right moisture and granulometry for their pelletizing. Furthermore, one of the aims of this research was to assess how suitable raw materials are for this process.

Three different pelletizing assays (P1–P3) were carried out from the raw materials in the study (W1–W3), as is shown in Table 1.

**Table 1:** Pelletizing (P1–P3) assays applied to raw materials.

Raw materials	P1	P2	P3
Cork powder 0.5–1 mm	100%	0%	0%
Cork powder 1–2 mm	0%	100%	0%
Cork powder 2–3 mm	0%	0%	100%

After that, five different mixtures (W4–W8), at different ratios, were carried out, as can be seen in Table 2. For mixing, an industrial balance (Adam, GFK series, Adam Equipment Co. Ltd., Kingston, UK) was used. For each case, the wastes were weighted, so that the right ratio was achieved, then they placed into a big bag and mixed by a manual tool until a uniform distribution was obtained.

**Table 2:** Pelletizing assays in waste mixtures.

Raw materials	P4	P5	P6	P7	P8
Cork powder 0.5–1 mm	50%	60%	40%	33%	50%
Cork powder 1–2 mm	50%	40%	60%	33%	0%
Cork powder 2–3 mm	0%	0%	0%	33%	50%

### 2.4. Pelletizing

Densification is based on material compacting in order to obtain denser products, with high heat power and homogeneity when it comes to their properties and size, replacing the main drawbacks related to draw materials (low bulk density, transportation difficulties, *etc.*). Among several densification techniques, pelletizing was selected for this study, carrying out a granulation in the product through extrusion.

**Figure 2:**
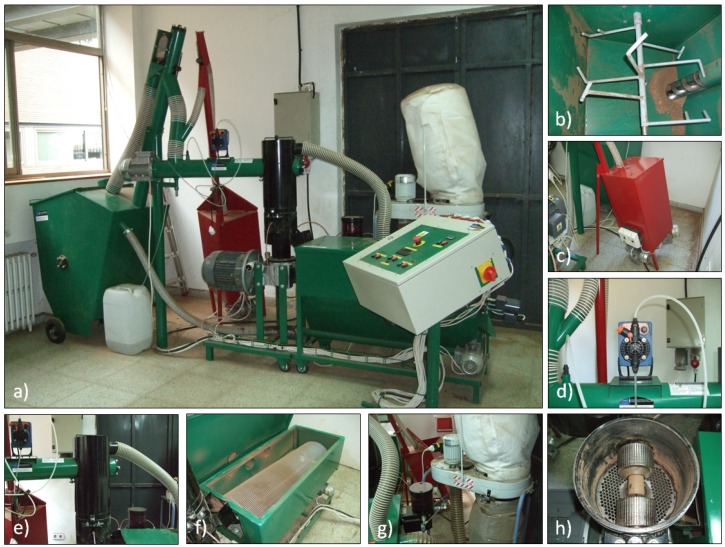
(**a**) Pelletizing equipment; (**b**) product hopper; (**c**) hopper and endless screw for auxiliary products; (**d**) water addition system; (**e**) flat die group; (**f**) cooling container; (**g**) lubrication and vacuum cleaner systems; and (**h**) flat die and rollers.

The obtaining of the pellet was carried out by a pelletizer with rotative flat die (Kovo Novak, MGL 200, Citonice, Czech Republic). The flat die is a horizontal disk with 6 mm-diameter holes. The rollers turn around their longitudinal axis on the flat die’s surface, generating high pressure on the product and forcing it through the holes, making its compaction possible. Figure 2 shows different images of the equipment in use.

In order to fix the tightening torque between the flat die and the rollers, a dynamometric key (Stahlwille 730N/10, Stahlwille Group, Wuppertal, Germany) was used, achieving 20 N·m. The mass flux of the densified product was 30 g/s, the amount that was used in previous assays, making sure to avoid obstructions during the process.

Temperature monitoring on the surface of the flat die’s frame was carried out by a thermographic camera (Flir T620, Flir Commercial Systems, Portland, OR, USA).

### 2.5. Densified Products Characterization

**Figure 3:**
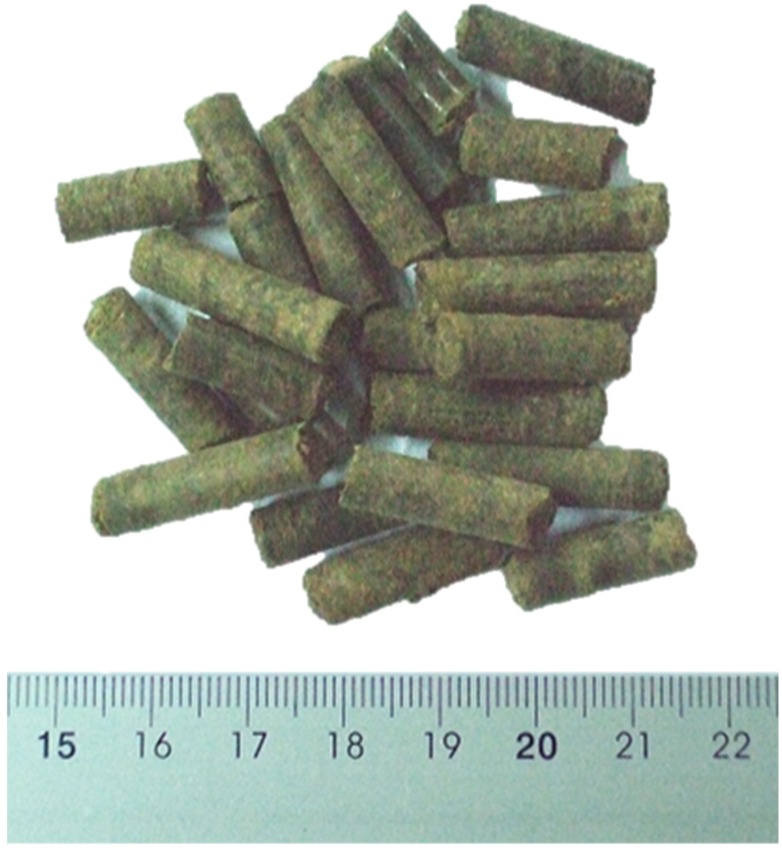
Pellets from the P2 assay.

Eight different types of pellet were produced (P1–P8). Figure 3 shows an example of the obtained pellets for one of the assays.

Pellets underwent different characterization assays. Thus, some intermediate samples were selected, rejecting both early and late pellets.

Densified product characterization was carried out following the standards in Table 1, finishing with other complementary measurements. Thus, average sizes (diameter and length) were determined by using a caliper and following the standard UNE-EN 16127 [22]. Durability assays were carried out according to the standard UNE-EN 15210-1 [23], using a durabilimeter (Mabrik BDM230, Mabrik, Barcelona, Spain), consisting of two rotatory boxes with smooth and flat surface that rotate at 50 rpm.

Finally, the results were compared to UNE-EN 14961-2 specifications [24], which establish three levels (A1, A2 and B, ordered from more to less demanding) for woody pellet commercialization for non-industrial use, so that quality is ensured for the final customer. In this study, the selected level (for further comparisons) was B, regarding recycled woody and industrial waste pellets. Moreover, the EN-Plus (B) quality certificate is based on this kind of pellet.

Characterization assays of the products and pellets were done in triplicate, taking the average as a valid value. In any case, the standard deviation was less than 3%.

## 3. Results and Discussion

### 3.1. Waste Characterization

Table 3 and Table 4 show the results for this study.

**Table 3:** Raw material characterization. W1, waste product 1. wb, wet basis; db, dry basis; *HHV*, high heating value; *LHV*, low heating value.

Characterization	W1	W2	W3
Moisture (%wb)	9.71	9.63	15.57
Bulk density (kg/m^3^ wb)	379.10	323.40	361.20
**Ultimate analysis**			
*C* (%db)	50.45	52.89	51.88
*H* (%db)	6.02	5.92	6.92
*N* (%db)	0.47	0.33	0.57
*S* (%db)	0.03	0.03	0.04
**Proximate analysis**			
Volatile matter (%db)	75.69	76.31	75.74
Fixed carbon (%db)	19.61	19.70	19.92
Ash content (%db)	4.70	3.99	4.34
*HHV* (MJ/kg db)	21.41	23.64	21.43
*LHV* (MJ/kg db)	20.08	22.33	19.90
*LHV* (MJ/kg wb)	18.13	20.18	16.81
Energy density (MJ/m^3^ wb)	6,873	6,526	6,072

**Table 4:** Physical characterization of waste combinations.

Characterization	W4	W5	W6	W7	W8
Moisture (%wb)	13.59	12.55	11.95	11.97	14.12
Bulk density (kg/m^3^ wb)	349.01	372.58	348.15	352.04	365.73

Moisture has a special influence on the densification process. Van Loo and Koppejan [25] claimed that moisture values in raw materials should be around 15%wb. Other authors, however, hold that 8%wb should not be surpassed [26]. On certain occasions, a higher percentage is necessary to make densification possible. Thus, Serrano *et al.* [27] obtained their best results when moisture values were in the range 19%wb–23%wb) for barley straw pellets.

Moisture content in the wastes in the study was less than 15%wb, obtaining higher values in some mixtures (compared to raw materials), possibly due to a higher environmental moisture during pelletizing.

**Figure 4:**
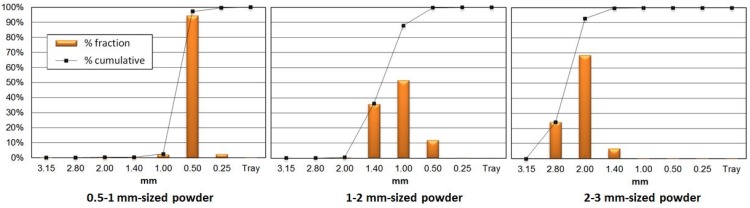
Granulometric analysis of original wastes.

Bulk density varied from 323 to 379 kg/m^3^ wb for the samples in the study. Generally, biomass wastes present low bulk densities on account of their porous structure, which makes their processing, storage and combustion difficult. When it came to granulometry, Figure 4 shows the particle distribution in raw materials. Most samples were located between three consecutive particles sizes, with 0.5, 1 and 2 mm as the major diameters for each case, these being lower than the maximum size recommended for pelletizing (3.15 mm) [28,29]. Therefore, the wastes in the study are suitable for direct pelletizing.

Regarding proximate analysis, high ash values were obtained. This fact will affect the combustion process directly, requiring ash removal more frequently, compared to other biofuels. 

### 3.2. Densified Products Characterization

Table 5 shows the obtained results after the characterization of the produced pellets.

**Table 5:** Characterization of pelletized products.

Characterization	P1	P2	P3	P4	P5	P6	P7	P8
Moisture (%wb)	8.02	7.96	6.53	7.37	8.41	8.11	7.89	9.04
Bulk density (kg/m^3^ wb)	697.02	705.10	698.54	692.35	689.18	687.64	688.08	686.57
Ratio of densification	1.84	2.18	1.93	1.98	1.85	1.98	1.95	1.88
Ultimate Analysis								
*C* (%db)	50.50	52.97	54.52	51.69	52.61	55.09	54.36	53.92
*H* (%db)	5.80	6.15	6.61	6.98	7.16	7.75	7.19	6.54
*N* (%db)	0.43	0.38	0.75	0.52	0.52	0.54	0.51	0.74
*S* (%db)	0.03	0.02	0.00	0.02	0.02	0.00	0.01	0.00
Proximate Analysis								
Volatile matter (%db)	78.78	78.71	75.15	76.14	77.55	77.47	76.33	77.81
Fixed carbon (%db)	19.41	17.16	20.11	20.03	18.21	18.51	19.57	18.08
Ash (%db)	4.81	4.13	4.74	3.84	4.24	4.02	4.10	4.11
*HHV* (MJ/kg db)	21.41	24.21	21.56	21.85	22.12	20.93	21.68	21.61
*LHV* (MJ/kg db)	20.13	22.85	20.10	20.31	20.54	19.22	20.09	20.17
*LHV* (MJ/kg wb)	17.72	21.03	18.79	18.81	18.81	17.66	18.51	18.35
Energy density (MJ/m^3^ wb)	12,351	15,569	13,126	13,785	12,966	12,144	12,734	12,595
Dimensions								
Length (mm)	24.51	24.12	24.47	25.95	23.94	25.09	24.86	24.05
Diameter (mm)	5.88	5.96	5.90	5.88	5.87	5.84	5.95	5.86
Durability (%)	96.79	97.17	97.67	98.36	98.24	97.62	97.69	97.38

**Figure 5:**
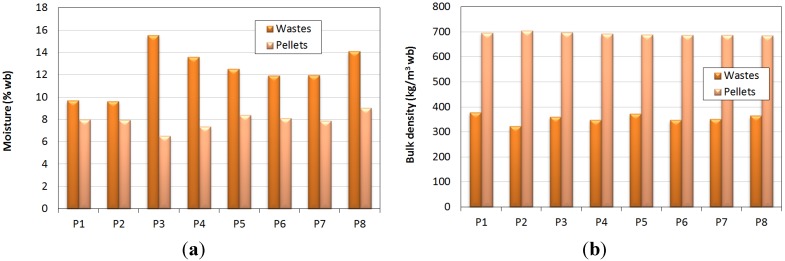
(**a**) Moisture in wastes and pellets; (**b**) bulk density in wastes and pellets.

With the properties of rejection powder being analogous, except for granulometric distribution, the pellet characteristics were also quite similar. Thus, Figure 5 sums up, graphically, the moisture and bulk density changes in the wastes in the study after pelletizing.

Moisture reduction is due to the temperature increase as a consequence of friction between the rollers and the flat die, with product heating and the consequent and partial water evaporation [12]. This decrease might be influenced by the initial moisture in raw materials, along with other variables that are difficult to assess, such as work rate, the pressure between flat die and rollers, *etc.* In our assays, the produced pellets presented moisture vales between 6.5%wb and 9%wb, despite the differences that were found in raw materials, especially when they were mixed (15%wb). This could be due to the fact that the achieved temperature in the flat die is so high that it could evaporate most of the water content in the wastes, obtaining quite similar moisture levels in densified products. At the same time, the higher moisture levels could be due to a higher production rate. Thus, raw material could have gotten through the flat die’s holes faster, and therefore, its contact with the hot matrix could be shorter, making evaporation milder compared to other products that could have spent longer periods in this stage.

Once the process was stabilized, the temperature obtained by the thermographic camera on the surface of flat die’s frame varied from 70 to 75 °C, keeping it constant until the end of the assays. No significative changes were observed depending on the raw material in use, and the slight humidity variations were on account of the different humidity levels of the wastes and mixtures in the study.

Thus, on account of the low initial moisture in the raw materials (under 15%wb), the registered percentages found in produced pellets were under 10%wb, being indispensable for a high-quality pellet certification. The achieved values were similar to those obtained by Nunes *et al.* [11] for pellets from equivalent residues (8%wb).

As far as bulk density is concerned, wastes and their mixtures showed slightly different values (between 323 and 379 kg/m^3^ wb), mainly due to the granulometric distribution, with the presence of holes or interstices depending on large-sized particles. Nevertheless, pellet density was quite similar, with an average value at around 693 kg/m^3^ wb. This observation could be due to the fact that, during pelletizing, particles are ground by the rollers and molded by the holes in the flat die, suppressing the interstice effect and being uniform in shape and structure.

**Figure 6:**
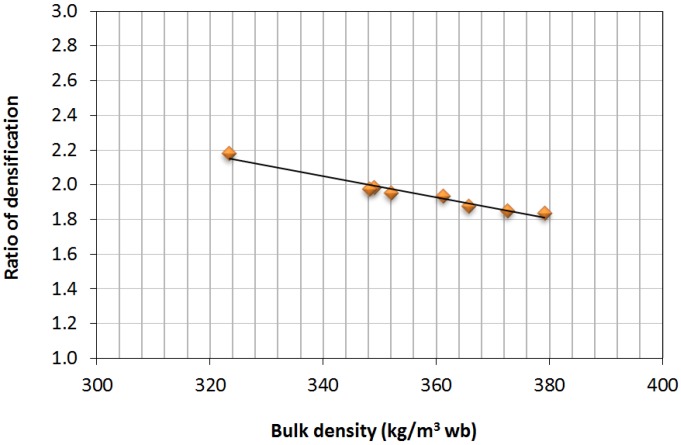
Comparison between bulk density and ratio of densification.

Bulk density in the densified products was higher, in all of the cases in the study, than the minimum that is demanded in standard EN-Plus (B), that is 600 kg/m^3^ wb, although another value (used in the design of woody biomass producers), that is 650 kg/m^3^ wb, is usually used [30]. Likewise, the observed densified ratio showed a volume reduction in the product as a consequence of pelletizing and, therefore, an increase in bulk density. Figure 6 shows the correlation between both variables according to obtained data.

The densified ratio values were between 1.84 and 2.18, showing how low initial densities in the raw material (or mixture) allowed for achieving higher compaction values. The obtained values were similar to those registered for oak tree pellets [31].

Regarding ultimate analysis, the pellets in the study showed low *S* and *N* levels (under 0.02% and 0.8%db, respectively), not predicting corrosion problems in boilers by sulfur or nitrogen oxide emissions [32].

Proximate analysis showed the same problems compared to granulometric separation powder used in this study. Thus, ash content was over 3%db, with values between 3.84%db and 4.24%db. The increase in temperature during the pelletizing process caused a decrease or release of certain products, and indirectly, the ash content increased [33]. Because of that, densified products showed less fixed carbon percentage and more ash content, respectively, than the raw materials. This situation was also observed during barley straw [27], Pyrenean oak, olive pomace and grape pomace pelletizing [12].

Other authors, such as Nunes *et al.* [11], obtained lower ash content in pellets from cork residues (between 2.5%db and 2.8%db), whereas Mediavilla *et al.* [13] registered values at around 4.4%db, slightly higher than those in this study.

Lehtikangas [33] carried out a study about the main characteristics of sawdust, woody biomass and pine bark, observing that ash content for the two latter (2.63%db and 3.71%db) was much higher than pine bark ash content (0.45%db). Filbakk *et al.* [34] also analyzed ash content in pine wood and bark, along with their mixtures (5%, 10% and 30%). The results showed 0.47% ash content for wood pellets and 2.50% for bark pellets, with values between them for the mixtures. In all cases, the higher the bark percentage was, the higher the ash content that was found. This could be related to granulometric separation powder waste pelletization, with one of the main components being cork bark. 

Granulometric separation powder pellets showed high *HHV* values, between 20.93 and 22.12 MJ/kg db. In the same way, *LHV* values were above 16 MJ/kg (wb), which is the minimum specification in standard EN-Plus (B). Equally, these values were higher than those found for pine, eucalyptus and forest wastes (between 16.00 and 18.13 MJ/kg db) [35,36].

The pellet size was within the limits specified by standard EN-Plus (B), which demands pellet diameter values between 6 ± 1 mm and length values between 3.15 and 40 mm. Concerning the length/diameter ratio (*L/D*), this is an important parameter, especially in pneumatic feed systems, due to the risk of obstruction in transportation pipes [30]. Even though standard EN-Plus does not define any restriction about it, other norms, such as the Österreichische Norm ÖNORM M 7135 [37], from Austria, establish that this ratio should be below five. The produced pellets satisfied this specification, with values between 4.08 and 4.41.

**Figure 7:**
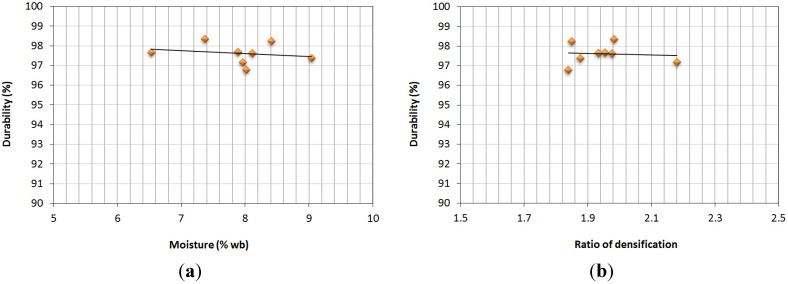
(**a**) Moisture, durability; (**b**) ratio of densification, durability.

Mechanical durability measures the effectiveness of the densification process, determining the quality of the product when it arrives to the final customer, being at once an important magnitude for the safety level. Dust generated as a consequence of efforts related to storage, transportation and later manipulation of densified products might imply serious risks for health, due not only to explosive atmosphere formation [33], but because it might cause respiratory diseases inn workers in the pelletizing industry or customers [38]. According to the assays in this research, Figure 7 shows the ratio between durability and two other properties in the study: moisture and the ratio of densification.

In both cases, it could be said that there was no defined correlation between the variables in the study. Generally, high moisture content in densified products (due to a high initial moisture in the raw materials or excessive water addition during pelletizing) implies defective agglomeration [27]. For the pellets in the study, there was no defective agglomeration, due to the negligible deviation among the analyzed values. Similar results were obtained by Miranda *et al.* [31], with 98.8%, 94.7% and 95.3% durability for forest biomass with 8.4%, 5.6% and 6.3% moisture (wb). Therefore, it could be said that slight changes in moisture do not affect the durability of densified products.

Equally, it can be observed that higher compacting levels do not imply higher durability or resistance to hits and pressure. Thus, Mediavilla *et al.* [13] registered 98.8% and 98.2% durability values for vine shoot and pine sawdust, with a 2.6 and a 3.1 ratio of densification, respectively, where higher ratios of densification did not imply high mechanical durability. The results of the assays were satisfactory in any case, regardless of the ratio of densification obtained, with values over 97% and similar to those obtained for forest residues pellets [39]. These values guarantee the right reception of the densified product for customers.

Table 6 sums up a comparison between the obtained data and the limits observed in the EN-Plus (B) norm for woody biomass for non-industrial use.

**Table 6:** Comparison with standard EN-Plus (B).

Characterization	P1	P2	P3	P4	P5	P6	P7	P8	EN-Plus (B)
Moisture (%wb)	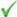	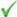	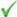	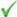	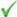	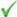	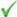	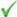	≤10
Bulk density (kg/m^3^ wb)	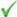	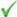	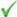	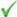	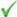	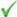	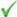	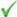	≥600
*N* (%db)	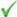	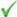	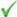	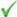	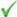	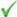	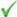	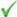	≤1.0
*S* (%db)	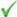	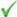	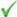	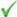	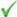	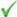	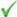	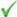	≤0.04
Ash (%db)	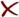	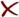	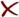	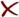	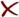	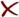	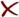	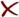	≤3.0
*LHV* (MJ/kg wb)	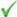	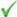	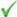	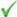	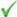	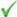	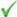	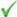	≥16
Length (mm)	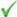	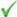	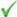	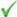	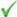	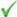	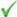	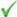	3.15–40
Diameter (mm)	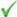	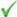	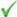	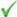	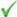	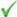	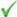	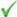	6 ± 1
Durability (%)	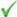	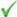	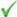	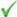	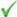	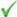	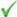	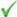	≥96.5

The results were satisfactory for all of the studied properties, except for ash content, whose value exceeded the allowed limit (3%db). For the rest of the variables, densimetric dust pellets went beyond the requirements in the compared norm. 

## 4. Conclusions

Cork powder is the generic name given to cork wastes in the industry, even though there are multiple differences depending on the stage and conditions of the process where they are generated. That is the reason why, in order to promote a specific use, a study about the possibilities of densimetric dust cork pelletizing (whose properties for thermal use clearly exceed those of the rest of the by-products in the cork industry) was carried out in this work. The most remarkable findings in this work were the following:

1. Pellets from granulometric separation powder satisfied all of the requirements in standard EN-Plus (B) for their use as a fuel for domestic application, except for ash content.
2. Granulometry changes were not a determinant factor for obtaining a densified product, on account of the slight differences between the different size distributions that were used in this study, being under 3.15 mm.
3. Pelletizing was not affected by the different moisture values in the wastes, due to the fact that they were within the recommended range (<15%wb), obtaining pellets with moisture under the allowed maximum in all cases (10%wb).
4. Different particle size mixing was not an obstacle during pelletizing process, and no pre-treatments (drying, sifting, *etc.*) were required in any case, with the consequent cost reduction related to the process.
5. Pellets from raw materials and the different mixtures in the study did not present significant differences regarding physical and energy characteristics. Thus, pelletizing might be dissociated from the real demand of granulated cork (0.5–1, 1–2 mm, *etc.*).

In future researches, the use of other types of wastes (such as those from the forest and wood industry) in cork mixtures will be studied, with the aim of reducing ash content under standard values for their use in boilers and domestic stoves. In the same way, ash composition and ash melting will be measured to give additional data about the densified biofuel.

## References

[1] ASECOR (2007). Cork: ecological, sustainable and recyclable. http://www.asecor.com/doc/elcorcho.pdf.

[2] Silva S.P., Sabino M.A., Fernandes E.M., Correlo V.M., Boesel L.F., Reis R.L. (2005). Cork: Properties, capabilities and applications. Int. Mater. Rev..

[3] Sepúlveda F.J. (2014). Selective Use for the Integral Valorization of Wastes from Cork Industry. Ph.D. Thesis.

[4] Gil L. (1997). Cork powder waste: An overview. Biomass Bioenergy.

[5] Rojas S., Pérez C., Montero I., Cobos J.G., De Miguel F. (2002). Energy recovery of waste from cork industries of San Vicente de Alcántara. Energía.

[6] Stelte W., Holm J.K., Sanadi A.R., Barsberg S., Ahrenfeldt J., Henriksen U.B. (2011). A study of bonding and failure mechanisms in fuel pellets from different biomass resources. Biomass Bioenergy.

[7] Sultana A., Kumar A., Harfield D. (2010). Development of agri-pellet production cost and optimum size. Bioresour. Technol..

[8] Nielsen N.P.K., Gardner D.J., Poulsen T., Felby C. (2009). Importance of temperature, moisture content, and species for the conversion process of wood into fuel pellets. Wood Fiber Sci..

[9] Holm J.K., Henriksen U.B., Hustad J.E., Sørensen L.H. (2006). Toward an understanding of controlling parameters in softwood and hardwood pellets production. Energy Fuels.

[10] Montero I., Miranda M.T., Sepúlveda F.J., Arranz J.I., Trinidad M.J., Rojas C.V. (2014). Analysis of pelletizing of wastes from cork industry. Dyna Energía y Sostenibilidad.

[11] Nunes L.J.R., Matias J.C.O., Catalão J.P.S. (2013). Energy recovery from cork industrial waste: Production and characterisation of cork pellets. Fuel.

[12] Arranz J.I. (2011). Analysis of Densified of the Combination from Different Biomass Waste. Ph.D. Thesis.

[13] Mediavilla I., Fernández M.J., Esteban L.S. (2009). Optimization of pelletisation and combustion in a boiler of 17.5 kWth for vine shoots and industrial cork residue. Fuel Process. Technol..

[14] (2010). UNE-EN 14772-2. Solid biofuels. Determination of moisture content.

[15] (2010). UNE-EN 15103. Solid biofuels. Determination of bulk density.

[16] (2011). UNE-EN 15149-2. Solid biofuels. Determination of particle size distribution.

[17] (2008). UNE-EN 15014. Plastics piping systems. Buried and above ground systems for water and other fluids under pressure.

[18] (2011). UNE-EN 15289. Solid biofuels. Determination of total content of sulfur and chlorine.

[19] (2010). UNE-EN 15148. Solid biofuels. Determination of the content of volatile matter.

[20] (2010). UNE-EN 14775. Solid biofuels. Determination of ash content.

[21] (2011). UNE-EN 14918. Solid biofuels. Determination of calorific value.

[22] (2012). UNE-EN 16127. Solid biofuels. Determination of length and diameter of pellets.

[23] (2010). UNE-EN 15210-1. Solid biofuels. Determination of mechanical durability of pellets and briquettes.

[24] (2012). UNE-EN 14961-2. Solid biofuels. Fuel Specifications and Classes.

[25] Van Loo S., Koppejan J. (2003). Handbook of Biomass Combustion and Cofiring.

[26] Granada E. (1999). Study of the Influence of the Parameters of Moisture, Temperature and Pressure at the Densification Process through Self-Agglomeration of Residual Lignocellulosic Biomass. Ph.D. Thesis.

[27] Serrano C., Monedero E., Lapuerta M., Portero H. (2011). Effect of moisture content, particle size and pine addition on quality parameters of barley straw pellets. Fuel Process. Technol..

[28] Mani S., Tabilb L.G., Sokhansanj S. (2006). Effects of compressive force, particle size and moisture content on mechanical properties of biomass pellets from grasses. Biomass Bioenergy.

[29] Wolf A., Vidlund A., Andersson E. (2006). Energy efficient pellet production in the forest industry—A study of obstacles and success factors. Biomass Bioenergy.

[30] Obernberger I., Thek G. (2004). Physical characterisation and chemical composition of densified biomass fuels with regard to their combustion behaviour. Biomass Bioenergy.

[31] Miranda M.T., Arranz J.I., Rojas S., Montero I. (2009). Enerdy characterization of densified residues from Pyrenean oak forest. Fuel.

[32] Miranda M.T. (2005). Thermal Utilization of Biomass. Ph.D. Thesis.

[33] Lehtikangas P. (2001). Quality properties of pelletised sawdust, logging residues and bark. Biomass Bioenergy.

[34] Filbakk T., Jirjis R., Nurmi J., Hoibo O. (2011). The effect of bark content on quality parameters of Scots pine (Pinus sylvestris L.) pellets. Biomass Bioenergy.

[35] Nhuchhen D.R., Salam P.A. (2012). Estimation of higher heating value of biomass from proximate analysis: A new approach. Fuel.

[36] Mustelier N.L., Almeida M.F., Cavalheiro J., Castro F. (2012). Evaluation of pellets produced with undergrowth to be used as biofuel. Waste Biomass Valor..

[37] Österreichs Norm. (2000). ÖNORM M 7135. Compressed wood and compressed bark in natural state—Pellets and Briquettes. Requirements and test specifications.

[38] Gillespie G.D., Everard C.D., Fagan C.C., McDonnell K.P. (2013). Prediction of quality parameters of biomass pellets from proximate and ultimate analysis. Fuel.

[39] Temmerman M., Rabier F. (2008). Comparative study of durability test methods for pellets and briquettes. Biomass Bioenergy.

